# Genetic engineering of *Pseudomonas chlororaphis* GP72 for the enhanced production of 2-Hydroxyphenazine

**DOI:** 10.1186/s12934-016-0529-0

**Published:** 2016-07-28

**Authors:** Kaiquan Liu, Hongbo Hu, Wei Wang, Xuehong Zhang

**Affiliations:** State Key Laboratory of Microbial Metabolism, School of Life Sciences and Biotechnology, Shanghai Jiao Tong University, Shanghai, 200240 China

**Keywords:** *Pseudomonas chlororaphis* GP72, Phenazine-1-carboxylic acid, 2-Hydroxyphenazine, Non-scar deletion, Overexpression

## Abstract

**Background:**

The biocontrol strain *Pseudomonas chlororaphis* GP72 isolated from the green pepper rhizosphere synthesizes three antifungal phenazine compounds, 2-Hydroxyphenazine (2-OH-PHZ), 2-hydroxy-phenazine-1-carboxylic acid (2-OH-PCA) and phenazine-1-carboxylic acid (PCA). PCA has been a commercialized antifungal pesticide registered as “Shenqinmycin” in China since 2011. It is found that 2-OH-PHZ shows stronger fungistatic and bacteriostatic activity to some pathogens than PCA. 2-OH-PHZ could be developed as a potential antifungal pesticide. But the yield of 2-OH-PHZ generally is quite low, such as *P. chlororaphis* GP72, the production of 2-OH-PHZ by the wide-type strain is only 4.5 mg/L, it is necessary to enhance the yield of 2-OH-PHZ for its application in agriculture.

**Results:**

Different strategies were used to improve the yield of 2-OH-PHZ: knocking out the negative regulatory genes, enhancing the shikimate pathway, deleting the competing pathways of 2-OH-PHZ synthesis based on chorismate, and improving the activity of PhzO which catalyzes the conversion of PCA to 2-OH-PHZ, although the last two strategies did not give us satisfactory results. In this study, four negative regulatory genes *(pykF, rpeA, rsmE* and *lon*) were firstly knocked out of the strain GP72 genome stepwise. The yield of 2-OH-PHZ improved more than 60 folds and increased from 4.5 to about 300 mg/L. Then six key genes (*ppsA, tktA, phzC, aroB, aroD* and *aroE*) selected from the gluconeogenesis, pentose phosphate and shikimate pathways which used to enhance the shikimate pathway were overexpressed to improve the production of 2-OH-PHZ. At last a genetically engineered strain that increased the 2-OH-PHZ production by 99-fold to 450.4 mg/L was obtained.

**Conclusions:**

The 2-OH-PHZ production of *P. chlororaphis* GP72 was greatly improved through disruption of four negative regulatory genes and overexpression of six key genes, and it is shown that *P. chlororaphis* GP72 could be modified as a potential cell factory to produce 2-OH-PHZ and other phenazine biopesticides by genetic and metabolic engineering.

**Electronic supplementary material:**

The online version of this article (doi:10.1186/s12934-016-0529-0) contains supplementary material, which is available to authorized users.

## Background

The biocontrol strain *Pseudomonas chlororaphis* GP72, isolated from the green pepper rhizosphere, has broad-spectrum antifungal activity against many agricultural phytopathogens [1, 2]. This capability depends primarily on the following three phenazine compounds: 2-Hydroxyphenazine (2-OH-PHZ), 2-hydroxy-phenazine-1-carboxylic acid (2-OH-PCA) and phenazine-1-carboxylic acid (PCA) [1, 3]. According to previous research carried out by our group, in *P. chlororaphis* GP72, the enzyme PhzO catalyzed the conversion of PCA to 2-OH-PCA. And the 2-OH-PCA was then spontaneously decarboxylated to 2-OH-PHZ [1, 3]. PCA is an effective antifungal pesticide and was registered as “Shenqinmycin” in 2011 by the Ministry of Agriculture of China as a biologically synthesized fungicide, which is based on its effectiveness against specific phytopathogens and minimal toxicity toward humans, animals, and the environment [4]. In previous studies, however, 2-OH-PHZ showed stronger fungistatic and bacteriostatic activity than PCA toward some pathogens, such as *Gaeumannomyces. graminis var. tritici* which cause the take-all disease of wheat [5]. The take-all disease is one of the most important root diseases of wheat worldwide, and no resistant cultivars or effective chemical fungicides are available, it is important to develop biological agents to control the disease [6–9]. Therefore, it is necessary to enhance the yield of 2-OH-PHZ for its application in agriculture. Although it may be practical to synthesize phenazines by chemical methods, the yield is low [10], and toxic byproducts such as lead oxide, aniline, o-phenylenediamine, and azobenzoate are produced [11]. So the biocatalytic synthesis of 2-OH-PHZ using recombinant microorganisms provides an attractive alternative. Until now, 2-OH-PHZ has been produced primarily by *P. chlororaphis* with the exception of the strain *P. aurantiaca* PB-St2 [12]. *P. chlororaphis* GP72 is a 2-OH-PHZ producing strain, although the production by the wild type is only 4.5 mg/L [1], it is possible to improve the 2-OH-PHZ production largely for its industrial production by genetic engineering and regulation.

The mechanism of the phenazine biosynthesis pathway has already been elucidated. A gene cluster *phzABCDEFG* facilitates phenazine biosynthesis in *Pseudomonas* [13]. The enzymes coded by *phzABCDEFG* convert chorismate, the end product of the shikimate pathway, into PCA, which is the synthesis substrate of the phenazine derivatives [13, 14]. In recent years, many studies on the production of shikimic acid (SA), a very important intermediate of the shikimate pathway, which is used as substrate in the synthesis of oseltamivir phosphate (Tamiflu) by chemical methods, have been reported [15]. To enhance the production of SA in recombinant microorganisms, several strategies have been developed to enhance the shikimate pathway. The principal purpose of these strategies is to increase the availability of phosphoenolpyruvate (PEP) and erythrose 4-phosphate (E4P), the direct precursors of the shikimate pathway, through genetic alterations that redistribut the metabolic fluxes in the central metabolism [16]. Other methods include improved channeling of carbon toward SA through overexpression of shikimate dehydrogenase, transketolase, DHQ synthase, feedback-resistant DAHP synthases, and DHQ dehydrogenase (coded by *aroE, tktA, aroB, aroFGH*^*fbr*^ and *aroD*) [17]. Besides that, strains lacking the pyruvate kinase (coded by *pykA* and *pykF*) and strains overexpressing PEP synthase (coded by *ppsA*) have also been evaluated as means of increasing the intracellular availability of PEP [18–20]. All of these strategies may be used in enhancing the production of phenazines for the shikimate pathway was necessary in synthesis of phenazines.

In addition, in *Pseudomonas protegens*, the Lon protease, which is an ATP-dependent protease, reduces the stability of the GacA protein and stymies the expression of the Gac/Rsm signal transduction system [21]. The GacS/GacA two-component system, which is conserved in many Gram-negative bacterias, stimulates production of phenazines and other secondary metabolites in *P. chlororaphis* 30–84 [22]. According to Wang et al. [22], overexpression of the gene *rsmE*, one of the RNA-binding proteins, resulted in decreased phenazine production in *P. chlororaphis* 30–84. Also, in our previous research, a negative regulatory gene *rpeA* was insertionally mutated to construct strain GP72AN, which resulted in a fivefold increase of 2-OH-PHZ (24.6 mg/L). All of these negative regulators may be used to enhance the production of phenazines.

**Fig. 1:**
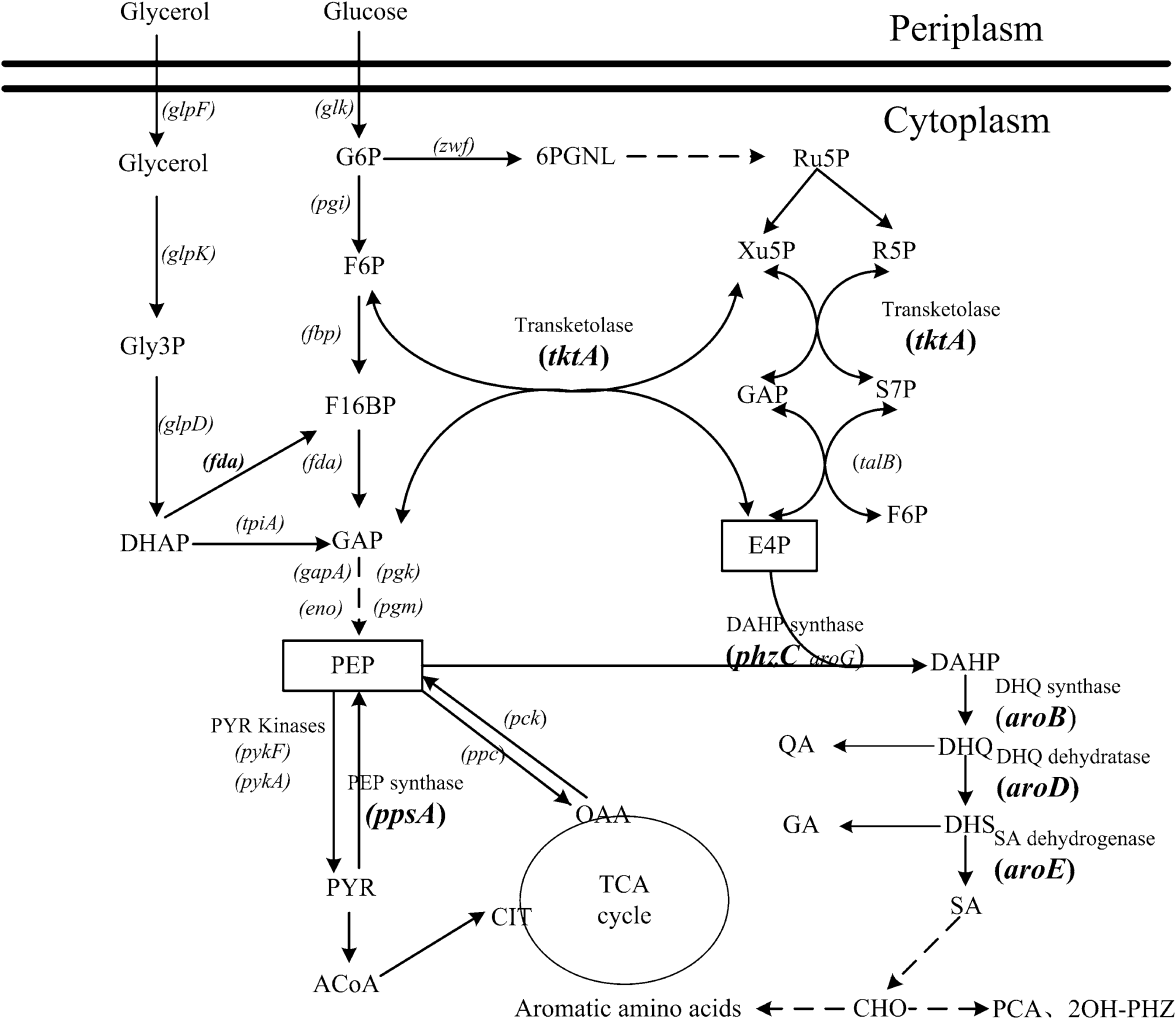
Genes selected from the gluconeogenesis, pentose phosphate and shikimate pathway to increase the phenazine yield. DHAP, dihydroxyacetone phosphate; Gly3P, Glycerol 3-phosphate; G6P, glucose 6-phosphate; F16BP, fructose 1,6-bisphosphate; GAP, glyceraldehyde 3-phosphate; F6P, fructose 6-phosphate; 6PGNL, 6-phosphogluconolactone; R5P, ribose 5-phosphate; Ru5P, ribulose 5-phosphate; S7P, sedoheptulose 7-phosphate; Xu5P, xylulose 5-phosphate; PEP, phosphoenolpyruvate; E4P, erythrose 4-phosphate; ACoA, acetyl-coenzyme A; PYR, pyruvate; OAA, oxaloacetate; CIT, citrate; DHQ, 3-dehydroquinic acid; DAHP, 3-deoxy-Darabinoheptulosonate7-phosphate; QA, quinic acid; DHS, 3-dehydroshikimic acid; SA, shikimic acid; GA, gallic acid; PCA, phenazine-1-carboxylic acid; CHO, chorismate; 2-OH-PCA 2-hydroxy-phenazine-1-carboxylic acid. Gene coding for enzymes not named in the figure: *pgi* phosphoglucose isomerase; *glk* glucokinase; *eno* enolase; *gapA* glyceraldehyde 3-phosphate dehydrogenase; *glpK* glycerol kinase; *glpF* glycerol facilitator; *fda* fructose-1,6-diphosphate aldolase; *glpD* glycerol-3-P dehydrogenase; *fbp* fructose 1,6-bisphosphatase; *tpiA* triosephosphate isomerase; *talB* transaldolase; *zwf* G6P dehydrogenase; *pck* PEP carboxykinase; *ppc* PEP carboxylase; *pgm* phosphoglyceromutase; *pgk* phosphoglycerate kinase

The aim of this study was to construct a genetically engineered *P. chlororaphis* GP72 strain to significantly increase 2-OH-PHZ production. Two aspects were investigated to achieve this goal: (1) the stepwise disruption of four negative regulatory genes (*pykF, rpeA, rsmE* and *lon*), and (2) the overexpression of six key genes (*ppsA, tktA, phzC, aroB, aroD* and *aroE*) selected from different pathways (Fig. 1). This work provides an efficient way for enhancing 2-OH-PHZ production.

## Results

### Disruption of four negative regulatory genes to enhance 2-OH-PHZ production

**Fig. 2:**
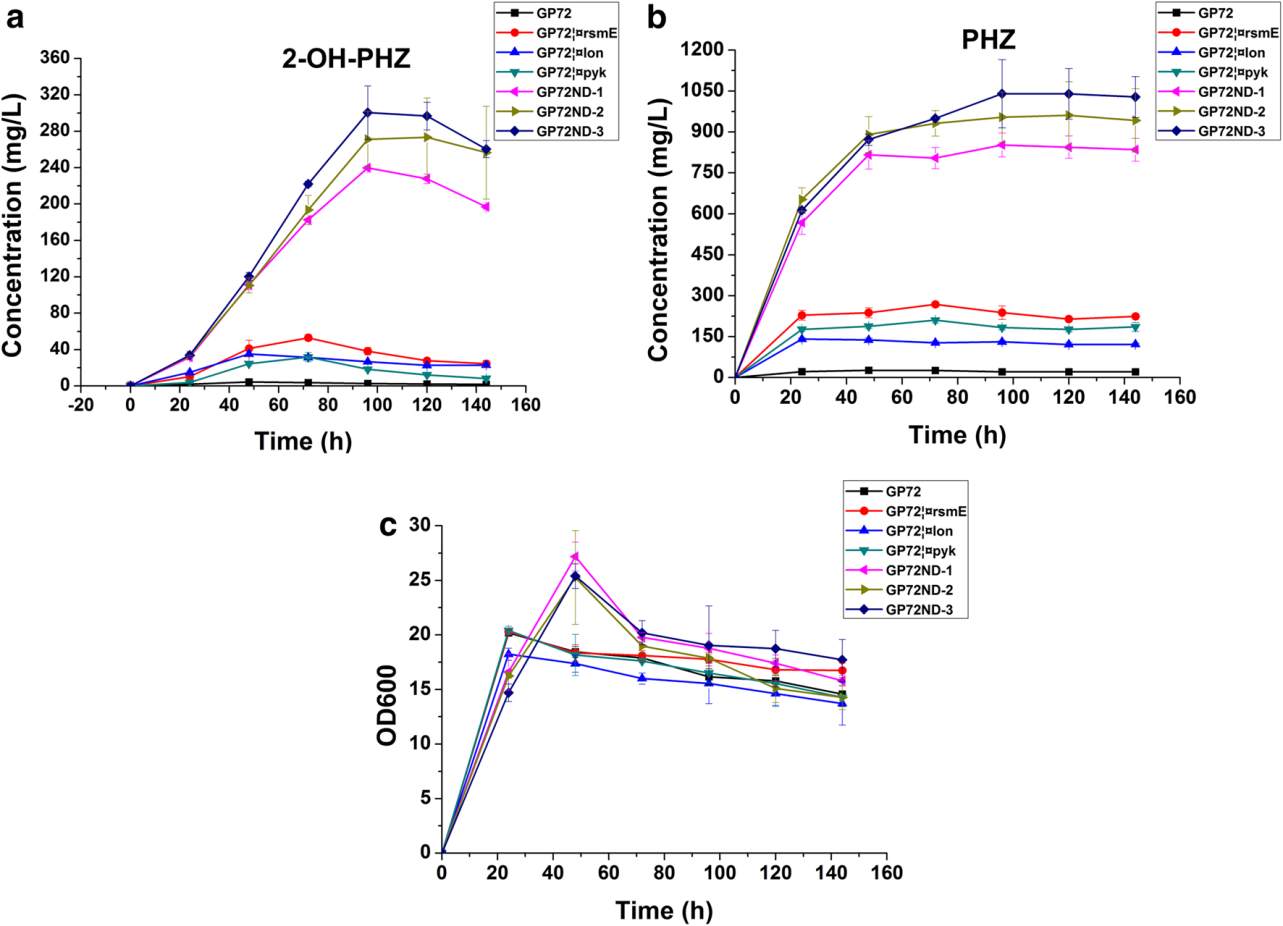
Growth curves, 2-OH-PHZ and PHZ production of the GP72 mutant derivative strains. **a** 2-OH-PHZ production. **b** Phenazine production. **c** Growth curves. The *error bars* indicate standard deviations from triplicate experiments

**Fig. 3:**
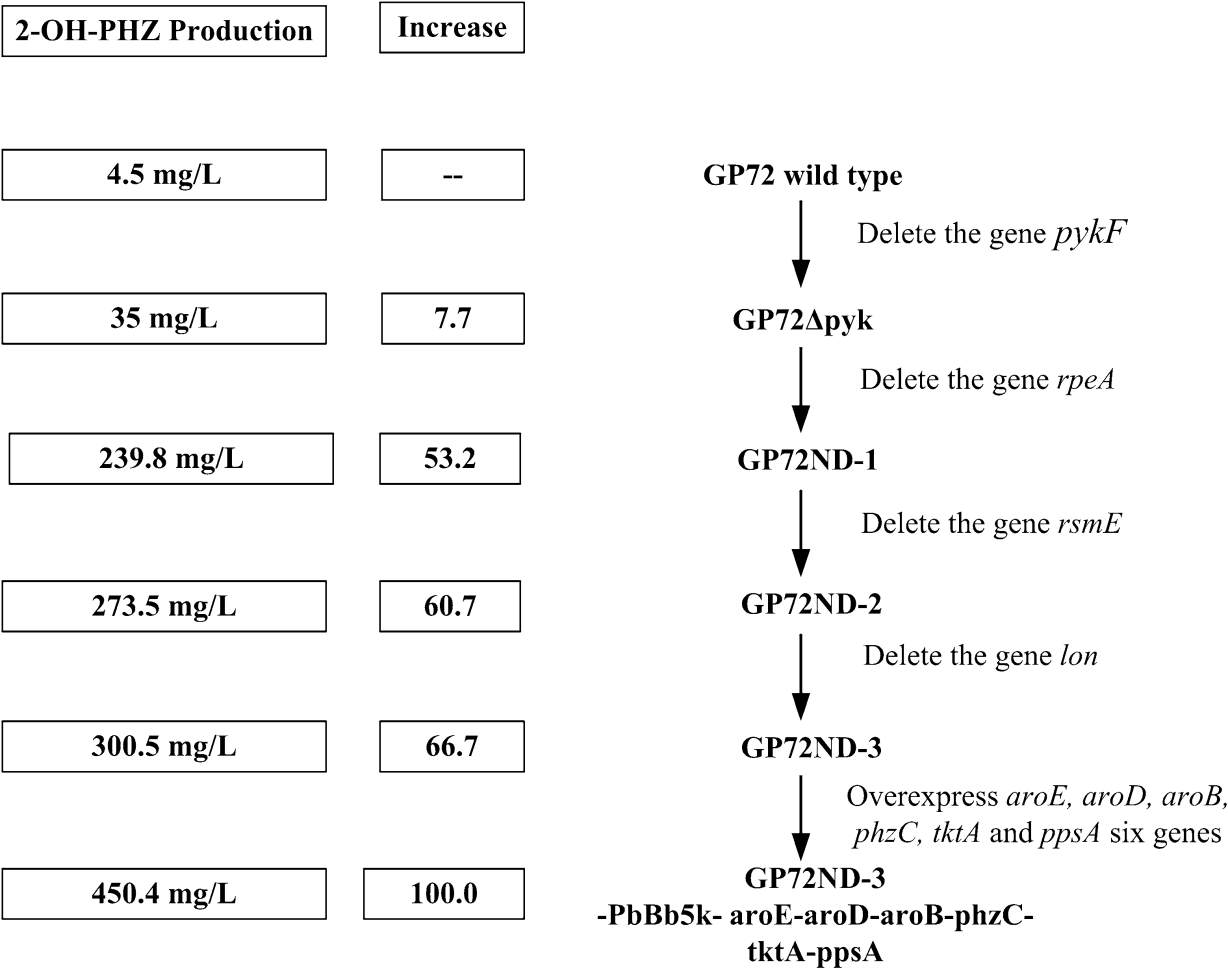
A summary of steps in the genetic and metabolic engineering of GP72 for 2-OH-PHZ production

In order to obtain a high yield of 2-OH-PHZ, we firstly chose to inactivate the gene *pykF* of GP72, and obtained the mutant strain GP72Δpyk (Figs. 2, 3). After fermentation and analyses by HPLC, the 2-OH-PHZ production of GP72Δpyk had a 6.7-fold increase compared to that of GP72, it increased from 4.5 to 35 mg/L (Figs. 2a, 3). And our results suggest that deletion of this gene had little effect on bacterial growth (Fig. 2c).

In this study, the strain GP72Δpyk was constructed with the non-scar deletion method (Additional file 1: Fig. S1). This is different from the insertional mutagenesis method that constructed GP72AN. GP72AN is a chromosomally inactivated GP72 mutant which was modified by inserting the resistance cassettes of gentamycin as a selective marker [1]. But insertional mutagenesis limited the multiplicity of genetic modification: *P. chlororaphis* GP72 is sensitive to only a few antibiotics [2]. Obviously, insertional mutagenesis could not be applied to inactivate the regulatory regions of GP72. Therefore, in this study, all of the mutant strains were constructed using the non-scar deletion strategy.

In our previous research, a negative regulator RpeA (coded by *rpeA*) was insertionally mutated to construct GP72AN, which resulted in a fivefold increase of 2-OH-PHZ [1]. In this work, we also knocked out *rpeA* from the GP72Δpyk genome to construct GP72ND-1 with the non-scar deletion strategy. Similar to the result of insertional mutagenesis, the 2-OH-PHZ production of GP72ND-1 had a 5.8-fold increase and reached 239.8 mg/L (Figs. 2a, 3).

RpeA belongs to the RpeA/RpeB two-component signal transduction system (TCST), and other strains of *Pseudomonas* also have homologues of RpeA [23]. For example, An RpeA homologue was found in *P. chlororaphis* 30–84 which negatively controlled the production of phenazine, indicating a conserved mechanism of phenazine regulation in *Pseudomonas* and other bacterias [24]. Other than RpeA/RpeB, GacS/GacA is the best known TCST system in *Pseudomonas* and is a master regulator of secondary metabolism, essential to phenazine production in *P. chlororaphis* and other *Pseudomonas* species [25, 26]. Studies have shown that GacA positively controls the expression of RsmX, RsmY, RsmZ, and other small non-coding RNAs which titrate the translational repressors RsmA and RsmE [26]. The contribution made by RsmA and RsmE to phenazine production was assessed in *P. chlororaphis* 30−84. Results indicated that RsmE is involved in the negative regulation of phenazines but RsmA is not [22]. Similar with *P. chlororaphis* 30–84, RsmA and RsmE were also identified in *P. chlororaphis* GP72. In order to enhance the yield of 2-OH-PHZ, the gene *rsmE* was knocked out of GP72 genome to construct GP72ΔrsmE. After HPLC analysis, the 2-OH-PHZ production of GP72ΔrsmE improved 11.7-fold (increased from 4.5 to 53 mg/L) compared with GP72 (Fig. 2a). This proved that *rsmE* should also have a negative regulatory role in the synthesis of 2-OH-PHZ in *P. chlororaphis* GP72. As a result, the gene *rsmE* was also knocked out of the strain GP72ND-1 genome to construct GP72ND-2, which resulted in the production of 2-OH-PHZ increasing from 239.8 to 273.5 mg/L (Figs. 2a, 3).

Most of bacterial intracellular proteolysis is initiated by four energy-dependent proteases: FtsH, HslUV, Lon, and the Clp family. Lon is key to over half of the energy-dependent proteolysis in *E. coli* [27]. According to a previous report, in *Pseudomonas protegens*, the Lon protease can impair the stability of GacA protein and reduce the expression of Gac/Rsm signal transduction pathway [21]. A *lon* gene knockout mutant has been previously shown to increase the production of antibiotics in *P. protegens* Pf-5 [28]. Importantly, the Gac/Rsm system is conserved in many Gram-negative bacterias, and it activates secondary metabolite production, including production of phenazines in *P. chlororaphis* 30–84 [22]. Thus, *lon* may participate in the regulation of phenazine synthesis. The gene *lon* was also found in *P. chlororaphis* GP72. We obtained the mutant strain GP72Δlon after the gene *lon* was knocked out of GP72, and the yield of 2-OH-PHZ had a 6.0-fold increase (from 4.5 to 31.7 mg/L) (Fig. 2a). In order to gain more 2-OH-PHZ, the gene *lon* was also knocked out from GP72ND-2 to construct GP72ND-3. After fermentation, the yield of 2-OH-PHZ increased from 273.5 to 300.5 mg/L (Figs. 2a and 3).

### Enhanced 2-OH-PHZ production by overexpressing six key genes

**Fig. 4:**
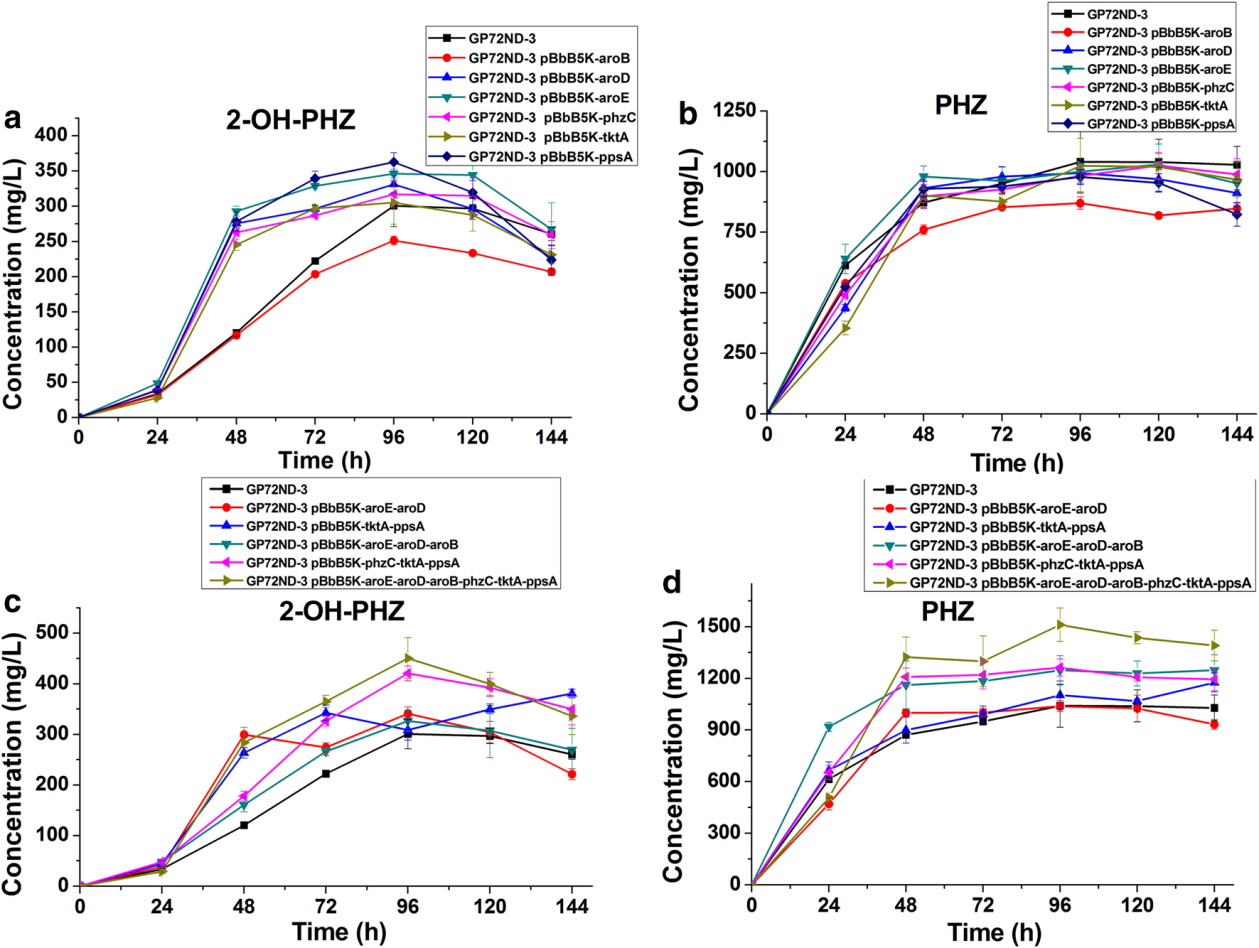
2-OH-PHZ and PHZ production in the mutimutant strain GP72-ND3 with different over-expressing plasmids. **a** 2-OH-PHZ production of different single genes overexpressing in GP72ND-3. **b** PHZ production of different single genes overexpressing in GP72ND-3. **c** 2-OH-PHZ production of different multiple genes overexpressing in GP72ND-3. **d** PHZ production of different multiple genes overexpressing in GP72ND-3. The *error bars* indicate standard deviations from triplicate experiments

Our results proved that the disruption of negative genes is a valid strategy for enhancing phenazines production. Compared with the disruption of negative genes, the overexpression of key genes is another effective strategy used frequently to enhance the production of biological products. As mentioned before, the disruption of *pykF* improved the production of 2-OH-PHZ by diverting more metabolic flux into the shikimate pathway. This suggested that enhancing the shikimate pathway may be a valid strategy to enhance the production of phenazines in GP72. In order to enhance the shikimate pathway of *P. chlororaphis* GP72, six key genes (*ppsA, tktA, phzC, aroB, aroD* and *aroE*) selected from the gluconeogenesis, pentose phosphate and shikimate pathways were chosen to be overexpressed. Because up to six genes would be simultaneously overexpressed, only a few kinds of antibiotics would be sensitive towards *P. chlororaphis* GP72 and be chosed to use [2]. To solve this problem, we chose a kind of modular vector—the BglBrick plasmid which has been recently widely used [19]. Firstly, six genes were constrcted into pBbB5K which is a BglBric plasmid. Then six genes were constructed into pbBb5K step by step, to build a fusion plasmid following the modular principle (Additional file 2: Fig. S2). To test the effect of overexpression, these pBbB5K plasmids were transformed into the strain GP72ND-3 by elec-transformation. The fermentation results showed that when single genes were overexpressed, the effect was not obvious (Fig. 4a, b). But when two or more adjacent genes were expressed at the same time, not only did 2-OH-PHZ production increase, but the production of other phenazines (such as PCA, 2-OH-PCA) also increased (Fig. 4c, d). When the fusion plasmid pBbB5K-aroE-aroD-aroB-phzC-tktA-ppsA was overexpressed in GP72ND-3, 2-OH-PHZ production of 450.4 mg/L (which increased 99-fold more than that of wide type GP72) and phenazine derivatives production of 1520 mg/L was obtained in the fermentation experiments (Figs. 3, 4). This result suggested that the overexpression of the corresponding genes was effective for enhancing 2-OH-PHZ production.

## Discussion

2-OH-PHZ, produced mainly by *P. chlororaphis*, is a potential antifungal pesticide, which has a broad application prospect. *P. chlororaphis* GP72 could produce 2-OH-PHZ, but its yield is only 4.5 mg/L. Therefore, it is necessary to enhance the 2-OH-PHZ production of GP72 for its application in agriculture. Here we tried four different strategies to improve the yield of 2-OH-PHZ, knocking out the negative regulatory genes, enhancing the shikimate pathway, deleting the competing pathways based on chorismate, and improving the activity of PhzO, although the last two strategies did not give us satisfactory results.

**Fig. 5:**
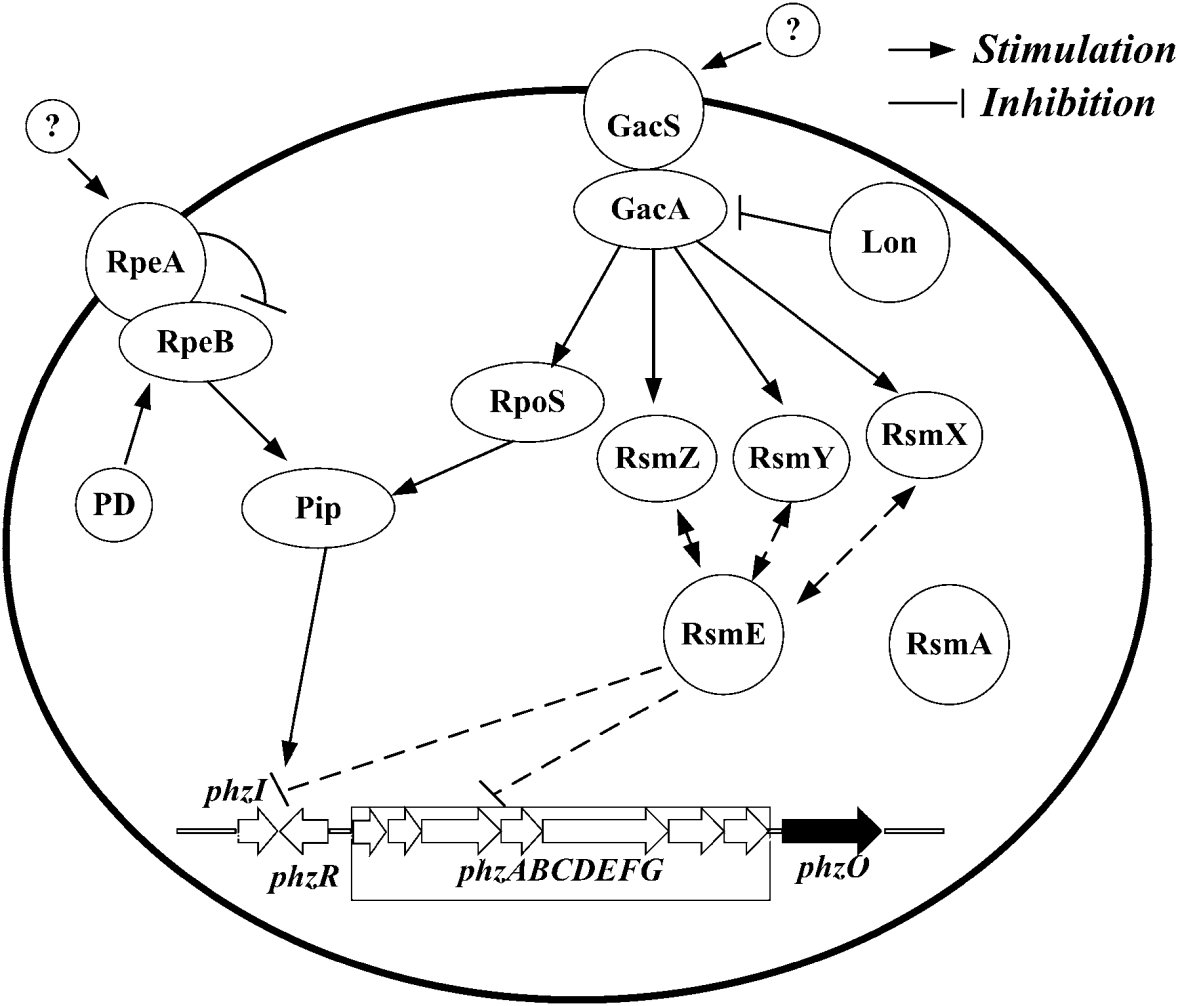
Proposed model for the regulation of phenazine biosynthesis by the TCST system in GP72. *Solid straight arrows* point to genes that are positively regulated. *Blunt lines* point to genes that are negatively affected. A *dashed arrow* indicates an unknown or as-yet uncharacterized regulatory pathway. In GP72, the sensors GacS and RpeB are activated by a putative environmental factor. Lon protease negatively affects GacA by controlling its protein stability. GacA positively controls the expression of rsmX, rsmY, and rsmZ, which in turn activates phenazine production by titrating the translation suppressor RsmE. In the absence of RpeA, RpeB is possibly over-phosphorylated by small phospho-donors (PD), resulting in the increased expression of the *pip*, *phzR*/*phzI* and the phenazine biosynthetic genes

In *P. chlororaphis*, the biosynthesis of phenazines has been traced to the shikimate pathway. This pathway begins with DAHP-synthase-mediated condensation of the central carbon metabolism intermediates erythrose 4-phosphate (E4P) and phosphoenolpyruvate (PEP) into 3-deoxy-d-arabinoheptulosonate 7-phosphate (DAHP). When PEP and E4P accumulate, it may channel the metabolic flow to biosynthetic routes of shikimate and chorismate, which are key metabolic precursors of phenazines. PEP is a key intermediate of the central metabolism, it serves as a precursor in several biosynthetic pathways [29]. Pyruvate synthesis from PEP catalyzed by pyruvate kinase (coded by *pyk*) is one of the main PEP consuming reactions [20]. Therefore weakening or blocking the conversion of PEP to pyruvate is a key strategy to enhance PEP availability (Fig. 1). According to previous research in *E. coli*, two pyruvate kinases were detected, which were coded by *pykA* and *pykF* respectively [30]. In contrast to the disruption of both *pyk* genes, a single inactivation of either the *pykA* or *pykF* genes may increase the quantity of PEP available for DAHP synthesis. This would not impede synthesis of pyruvate or the flux of pyruvate to acetyl-CoA [18]. It has been shown in *E. coli* that both *pykA* and *pykF* have active roles in pyruvate biosynthesis and PykF displayed the higher enzyme activity when compared to PykA in the wild type strain [30]. Therefore, PykF is thought to produce the main Pyk activity in *E. coli* [20]. Similar to *E. coli*, *P.chlororaphis* GP72 also contains two pyruvate kinases, which were coded by *pykA* and *pykF*. In order to increase the availability of the PEP, the gene *pykF* of GP72 was selected to knock out. And the 2-OH-PHZ production had a 6.7-fold increase (Figs. 2a, 3). TCST systems help prokaryotes interact with their environments through both sensing and response, coordinating many of cellular pathways. *Pseudomonas species* contain a large number of TCST systems, for example, there are 91 in *P. fluorescens* Pf-5, 127 in *P. aeruginosa* PAO1, and 51 in *P. chlororaphis* 30–84 [24]. In *P. chlororaphis* GP72, a large number of TCST systems also have been discovered. Huang et al. [1] have reported that RpeA negatively controlled the production of 2-OH-PHZ in *P. chlororaphis* GP72. In this study, three genes (*rpeA, rsmE* and *lon*), which are all related to the TCST systems, were knocked out of the strain GP72Δpyk genome stepwise. After delection of these three genes, the 2-OH-PHZ production increased from 35 to 300.5 mg/L. According to previous reports on *P. chlororaphis* 30–84, *P. chlororaphis* PCL1391 and our results, we propose a simple regulatory model of GP72 to illustrate the role of phenazine regulators modulated by TCST systems [1, 22, 24, 31] (Fig. 5). In this simple model, GP72 is similar to the strains *P. chlororaphis* PCL1391 and 30–84, Pip promotes the production of phenazine by increasing the expression of phzI and phzR. The expression of Pip is regulated by the sigma factor rpoS, which is itself regulated by GacA, and by the RpeA/RpeB TCST system. Each of the RpeA/RpeB and RpoS regulates phenazine production and both of them use pip as a regulatory intermediate. However, GacS/GacA and RpeA/RpeB do not regulate phenazines hierarchically. The GacS/GacA system also regulates the synthesis of phenazines through the noncoding RNAs (ncRNAs) pathway (including rsmX, rsmY, and rsmZ). GacA is involved in the positive controls of noncoding RNAs expression, which titrate the translational repressors RsmA and RsmE. And only RsmE, not RsmA, is involved in the negative regulation of phenazines. Lon protease (coden by *lon*) also regulates the phenazine synthesis through negatively affecting the stability of GacA protein.

**Fig. 6:**
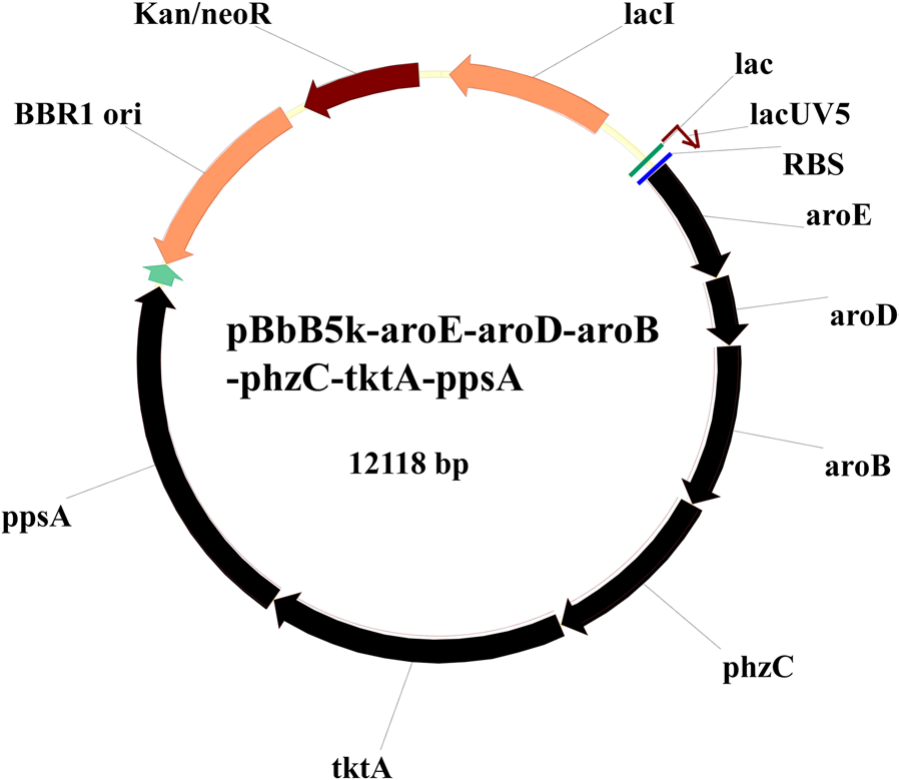
The BglBrick plasmid of pBbB5K-aroE-aroD-aroB-phzC-tktA-ppsA, which was overexpressed to enhance the production of 2-OH-PHZ. The gene order in the plasmid was *aroE*, *aroD*, *aroB*, *phzC*, *tktA* and *ppsA*, which is a reverse of the expression pathway

In this work, we tried to improve the 2-OH-PHZ production of GP72 by enhanceing the shikimate pathway. According to previous research, several successful strategies have been used to enhance the shikimate pathway in *E. coli* strains, for example, by increasing the availability of direct precursors PEP and E4P, improving DAHP synthesis in the shikimate pathway, enhancing the metabolic flow through the biosynthetic pathway by impeding allosteric and transcriptional regulation, and identifying and interfering with rate-limiting enzymatic reactions [32]. Disruption of pyruvate kinases is not the only way which could increase the availability of the direct precursors of shikimate pathway. Several other strategies have been reported. On the one hand, high PEP availability was achieved by improving the recycling of PYR to PEP by overexpression of gene *ppsA* (code PEP synthetase) [33]. On the other hand, high E4P availability could be achieved by the overexpression of gene coding for a transketolase (*tktA*) [34]. So *ppsA* (code PEP synthetase) and *tktA* (code transketolase) in GP72 were selected for overexpression to enhance shikimate pathway. Further increases in carbon flux through the shikimate pathway were realized by removing the allosteric and transcriptional regions and by relieving impeding enzymatic reactions [35]. The reactions catalyzed by DHQ dehydratase (coded by *aroD*), quinate/shikimate dehydrogenase (coded by *aroE*), dehydroquinic acid (DHQ) synthase (encoded by *aroB*) and DHAP synthetase (coded by *aroFGH* in *E. coli*) have been reported as limiting steps in the shikimate pathway [36]. Therefore, four key genes of GP72 *(phzC, aroB, aroD, aroE* which code DAHP synthase, DHQ synthase, DHQ dehydratase and SA dehydrogenase respectively) were also selected to enhance the shikimate pathway of GP72 (Fig. 1). Thus six key genes (*ppsA, tktA, phzC, aroB, aroD* and *aroE*) selected from different pathways were overexpressed to enhance the skimate pathway. According to previous report of Juminaga et al. [19], genes located near the promoter usually show much higher levels of induction than those distant from the promoter. The reverse arrangement of genes in the operon was found to benefit from the formation of the desired product through higher concentrations of protein in the latter part of the pathway. Therefore, we followed this method, in which the gene order in the fusion plasmid was aroE-aroD-aroB-phzC-tktA-ppsA, and the reverse order of the expression pathway (Fig. 6). When the plasmid pBbB5K-aroE-aroD-aroB-phzC-tktA-ppsA was overexpressed in GP72ND-3, the 2-OH-PHZ production increased to 450.4 mg/L (which was near 100-fold of that in the wide type GP72) and phenazine derivatives production also increased to 1520 mg/L.

Preventing the loss of carbon flow towards competing pathways is a successful strategy to enhance the production of phenazines [37]. *P. chlororaphis* GP72 synthesizes phenazines with the enzymes encoded by the *phz* cluster using chorismate as a precursor (Fig. 1). GP72 contains at least four additional chorismate-utilizing pathways for the synthesis of folate, tryptophan, co-enzyme Q, tyrosine and phenylalanine. The phenazine biosynthetic pathway must compete with these pathways for chorismate, which is a central intermediate precursor. In order to enhance the production of phenazines, we tried to block these chorismate-utilizing pathways. Thus, *pabB*/*pabC* encoding para-aminobenzoate synthase, *trpE* encoding anthranilate synthase, and *pheA* encoding chorismate mutase/prephenate dehydratase were all deleted from GP72. The attempt was not successful because of the bad growth of these mutants (unpublished data), although a successful precedent was obtained in *Pseudomonas aeruginosa* PA1201 [37]. Therefore, in this work the genetic modification of GP72 mainly focused on the regulatory genes.

The phenazines produced in *P. chlororaphis* GP72 include 2-OH-PHZ, 2-OH-PCA and PCA [1]. According to our previous research on *P. chlororaphis* GP72, 2-OH-PHZ was derived from PCA, through the action of the enzyme, PhzO [1, 3]. Differing from other *P. chlororaphis* strains, PCA could not be completely converted to 2-OH-PHZ by PhzO in the GP72 strain because of the high production of PCA [1–3]. Previous studies have shown the production of 2-OH-PCA in *P. chlororaphis* GP72AN to be relatively low, only 10–20 % of PCA [3]. Improving the efficiency of PhzO, which converts PCA to 2-OH-PHZ is also a strategy for enhancing 2-OH-PHZ production, although the mechanism to improve the activity of PhzO is still unclear. In this study, we analyzed the codon usage of *phzO*. Many rare codons were found to exist in this gene. To improve the expression of *phzO*, rare codons of the *phzO* were optimized, yielding *phzO*^*op*^, the codon-optimized variant of *phzO*. The *phzO*^*op*^ was introduced into the GP72 and replaced the *phzO* gene successfully, generating corresponding strains. However, according to the fermentation result, optimization of the *phzO* gene did not improve the efficiency of PCA to 2-OH-PHZ conversion (unpublished data). This result suggested that the rare codon usage of *phzO* may not be the reason why PCA cannot be converted to 2-OH-PHZ completely. Currently, a study on promoting the conversion of PCA to 2-OH-PHZ is in progress.

In this work, four regulatory genes *pykF, rpeA, rsmE* and *lon* were firstly knocked out of the strain GP72 genome step by step. Then, up to six genes of *ppsA, tktA, phzC, aroB, aroD* and *aroE* were constructed into one BglBrick vector-pBbB5K and overexpressed to enhance the production of phenazine derivatives. A strain producing 450.4 mg/L of 2-OH-PHZ and 1520 mg/L of phenazine derivatives was obtained. This study laid a good foundation for the future industrial production and agricultural application of biopesticide phenazines.

## Conclusions

In this work, four regulatory genes *pykF*, *rpeA,rsmE* and *lon* were firstly knocked out of the strain GP72 genome step by step. Then, *ppsA*, *tktA*, *phzC*, *aroB*, *aroD* and *aroE* six genes were constructed into one BglBrick vector-pBbB5K and overexpressed to enhance the production of phenazine derivatives. A strain producing 450.4 mg/L of 2-OH-PHZ and 1520 mg/L of phenazine derivatives was obtained. This study laid a good foundation for the future industrial production and agricultural application of biopesticide phenazines.

## Methods

### Bacterial strains, plasmids and growth conditions

The bacterial strains, plasmids and primers used in this study are listed in Table 1 and Additional file 3: Table S1. *Escherichia coli* was cultured in Luria–Bertani (LB) medium (Tryptone 10 g, Yeast extract 5 g, NaCl 10 g/L) at 37 °C. *P. chlororaphis* GP72 and its derivative strains were grown in LB or King’s medium B (glycerol 15 mL, tryptone 20 g, MgSO_4_ 0.732 g, K_2_HPO_4_ 0.514 g/L) at 28 °C. Antibiotics in the medium were used at the following concentrations: Ampicillin (Ap, 100 μg/mL), Gentamicin (Gm, 50 μg/mL) and Kanamycin (Kn, 50 μg/mL).

**Table 1:** Strains and plasmids used in this study

Strains and plasmids	Relevant gene type	Reference/source
Strains		
DH5α	*E. coli* F^−^Ф80*lacZ*ΔM15 Δ(*lacZYA*-*argF*) U169 *recA1* *endA1* *hsdR17* (r_k_^−^ m_k_^−^) *phoA* *supE44* *thi* ^−1^ *gyrA96* relA1	Lab stock
* E.coli* S17-1 (λpir)	res^−^ pro mod^+^ integrated copy of RP4, mob^+^, used for incorporating constructs into *P. chlororaphis*	Hoffmann et al. [38]
GP72	*P. chlororaphis* GP72 wild-type strain	Lab stock
GP72AN	*rpeA* insertionally inactivation mutat of GP72	Lab stock
GP72Δpyk	*pykF* in-frame deletion mutant of GP72	This study
GP72ΔrsmE	*rsmE* in-frame deletion mutant of GP72	This study
GP72Δlon	*lon* in-frame deletion mutant of GP72	This study
GP72ND-1	*rpeA* in-frame deletion mutant of GP72Δpyk	This study
GP72ND-2	*rsmE* in-frame deletion mutant of GP72ND-1	This study
GP72ND-3	*lon* in-frame deletion mutant of GP72ND-2	This study
GP72ND-3-pBbB5K-aroB	*aroB* overexpression in GP72ND-3	This study
GP72ND-3-pBbB5K-aroD	*aroD* overexpression in GP72ND-3	This study
GP72ND-3-pBbB5K-aroE	*aroE* overexpression in GP72ND-3	This study
GP72ND-3-pBbB5K-phzC	*phzC* overexpression in GP72ND-3	This study
GP72ND-3- pBbB5K-tktA	*tktA* overexpression in GP72ND-3	This study
GP72 ND-3- pBbB5K-ppsA	*ppsA* overexpression in GP72ND-3	This study
GP72 ND-3- pBbB5K-aroE-aroD	*aroE and aroD* overexpression in GP72ND-3	This study
GP72 ND-3-pBbB5K-aroE-aroD-aroB	*aroE, aroD and aroB* overexpression in GP72ND-3	This study
GP72 ND-3- pBbB5K-tktA-ppsA	*tktA* and *ppsA* overexpression in GP72ND-3	This study
GP72 ND-3- pBbB5K-phzC-tktA-ppsA	*phzC, tktA* and *ppsA* overexpression in GP72ND-3	This study
GP72 ND-3- pBbB5K-aroE-aroD-aroB-phzC-tktA-ppsA	*aroE, aroD, aroB, phzC, tktA* and *ppsA* overexpression in GP72ND-3	This study
Plasmids		
pMD19-T simple	T-Vector for gene coloning, Ap^r^	Lab stock
pEASY-Blunt	Blunt vector for gene coloning, Ap^r^, Kan^r^	Lab stock
pMD19-T-aroD	Site-specific mutant vector for gene *aroD*	This study
pEASY-Blunt-tktA	Site-specific mutant vector for gene *tktA*	This study
pEASY-Blunt-ppsA	Site-specific mutant vector for gene *ppsA*	This study
pK18mobsacB	Broad-host-range gene replacement vector, *sacB*, Kan^r^	Schafer et al. [39]
pK18-pyk	pK18mobsacB containing *pykF* flanking region	This study
pK18-rsmE	pK18mobsacB containing *rsmE* flanking region	This study
pK18-lon	pK18mobsacB containing *lon* flanking region	This study
pBbB5K-GFP	pBBR1; Kn^r^ lacI P_lac-UV5_	Lee et al. [40]
pBbB5K-aroB	Plasmid for *aroB* overexpression	This study
pBbB5K-aroD	Plasmid for *aroD* overexpression	This study
pBbB5K-aroE	Plasmid for *aroE* overexpression	This study
pBbB5K-phzC	Plasmid for *phzC* overexpression	This study
pBbB5K-tktA	Plasmid for *tktA* overexpression	This study
pBbB5K-ppsA	Plasmid for *ppsA* overexpression	This study
pBbB5K-aroE-aroD	Plasmid for *aroE,* *aro*D overexpression	This study
pBbB5K-aroE-aroD-aroB	Plasmid for *aroE,* *aro*D *and aroB* overexpression	This study
pBbB5K-tktA-ppsA	Plasmid for *tktA* and *ppsA* overexpression	This study
pBbB5K-phzC-tktA-ppsA	Plasmid for *phzC, tktA* and *ppsA* overexpression	This study
pBbB5K-aroE-aroD-aroB-phzC-tktA-ppsA	Plasmid for *aroE,* *aroD, aroB,phzC, tktA* and *ppsA,* overexpression	This study

### Construction of the non-scar deletion mutant strains

Two pairs of primers, lonF1 (EcoRI)-lonR1 and lonF2-lonR2 (XbaI), were designed to delete the *lon* sequence from *P. chlororaphis* GP72. Then PCR was used to amplify a 700 bp DNA fragment covering the upstream area of the *lon* gene and an 820 bp DNA fragment covering the downsteam of the *lon* gene. Overlap PCR was performed to align the two fragments, which shared a 16 bp homologous region, as shown in Additional file 1: Fig. S1. The 1.5 kb DNA fragment that had not been modified with scars was digested with the restriction enzymes XbalI and EcoRI and cloned into the plasmid pK18mobsacB. The products were then transferred to *E. coli* S17-1 (λpir) and then moved to *P. chlororaphis* GP72 to generate the mutant GP72Δlon through biparental mating. Single-crossover clones were selected on plates containing 50 μg/mL Kn and 100 μg/mL Ap. Then double-crossover clones were selected on plates containing 15 % sucrose. These were confirmed by PCR analysis and sequencing. The simple sketch for constructing a non-scar deletion GP72Δlon is shown in Additional file 1: Fig. S1.

In similar ways, the *pykF, rpeA* and *rsmE* non-scar deleted mutants were constructed in their corresponding strains.

### DNA manipulations for overexpression

All genes were PCR-amplified from the genomic DNA of *P. chlororaphis* GP72 and extended by using the 5′ terminal sequence 5′-AAAGGAGGCCATCC-3′ and endonuclease restriction sites located at the 5′ and 3′ ends [19]. All of these genes were cloned into T vector pMD19-T or pEASY-Blunt and transformed into *E. coli.*

The plasmids used for overexpression were constructed following the Bglbrick standard, and the plasmid pBbB5KGFP was used as the backbone. All XhoI, BglII, BamHI, and EcoRI restriction sites were removed from all genes cloned into BglBrick plasmids (pBbB5K). Because the genes *aroD, tktA* and *ppsA* contain 1–3 of these restriction sites respectively, point mutation was used to remove these restriction sites. Point mutations were conducted using a Quick-Changes™ Site-Directed Mutagenesis Kit following the manufacturer’s instructions (La Jolla, CA, USA). The ORFs were then PCR-amplified using primers that extended the 5′ ends with EcoRIXXBglII, the consensus 5′-AGGAGG-3′ ribosome binding site (RBS) and a spacer sequence, 5′-CCATCC-3′ and the 3′ ends with BamHIXXXhoI. Like the other ORFs in this study, before cloning, all PCR fragments were digested with XhoI and EcoRI then inserted into the plasmid from the 5′ end to the 3′ end, replacing the gene *GFP* which contained in the plasmid originally [19].

In brief, six brick plasmids, which contain pBbB5K-aroB, pBbB5K-aroD, pBbB5K-aroE, pBbB5K-phzC, pBbB5K-tktA and pBbB5K-ppsA respectively, were first constructed. Subsequently, the overexpression plasmids which contained two, three or six genes were constructed. A simplified scheme of the steps used in the construction of these plasmids is shown in Additional file 2: Fig. S2.

All of the strains which held plasmids for over-expression were generated by transforming the plasmid into corresponding competent cells with elec-transformation (Bio-Rad, Hercules, USA).

### Fermentation processing

*Pseudomonas chlororaphis* GP72 and its derivative strains stored in a −80 °C freezer were activated at 28 °C for 12-24 h in King’s B agar media with the corresponding antibiotics. Selection of single colonies from Petri plates was performed, which were then used to inoculate approximately 5 mL of King’s B broth (supplemented with the corresponding antibiotics) in 50 mL flasks. Cultures were then incubated at 28 °C with 180 rpm of shaking overnight. Portions of these cultures were then inoculated into 250 mL baffled flasks containing 60 mL King’s B broth with corresponding antibiotics to achieve an initial OD600 of 0.02. Then the fermentation process was initiated. For induction of the overexpression strains, 50 μM IPTG (isopropyl-β-d-thiogalactopyranoside) was added to the culture after 12 h of incubation time. After 24–120 h growth at 28 °C and 180 rpm, cultures were collected for the measurement of phenazine compounds and OD600. Triplicate experiments were carried out for each fermentation test.

### Quantification of phenazine compounds

In order to quantify the phenazine compounds, the fermentation broth was firstly adjusted to pH 2.0 with 6 M HCl. Then the fermentation broth was extracted with three volumes of ethyl acetate with vigorous shaking. The organic layer was collected and mixed with a 1/10 volume of distilled water. The mixture was then shaken rigorously. Finally, the organic phase containing phenazine compounds was evaporated under vacuum pressure. The phenazine compounds were dissolved in methanol for further analysis. HPLC analysis was performed on the phenazine compounds (2-OH-PCA, 2-OH-PHZ, and PCA) using a 1260 Infinity HPLC apparatus (Agilent Technologies Group, Santa Clara, USA) with a C-18 reverse phase column and UV detector (Agilent, Santa Clara, USA) as described previously [1].

### Nucleotide sequence accession numbers

The nucleotide sequence of the genes used in this work were deposited in the GenBank, National Center for Biotechnology Information, http://www.ncbi.nlm.nih.gov/nucleotide. They correspond to the genome shotgun sequence of *P. chlororaphis* strain GP72, more information was shown in Additional file 4: Table S2.

## Additional files

10.1186/s12934-016-0529-0 Strategy used for non-scar gene deletion.
10.1186/s12934-016-0529-0 Simplified scheme of the steps required in the construction of the BglBric plasmid pBbB5K-aroE-aroD-aroB-phzC-tktA-ppsA.
10.1186/s12934-016-0529-0 Primers used in this study.
10.1186/s12934-016-0529-0 The accession number of genes used in this work were deposited in GenBank of NCBI.

## Abbreviations

2-OH-PCA: 2-hydroxy-phenazine-1-carboxylic acid
2-OH-PHZ: 2-Hydroxyphenazine
DAHP: 3-deoxy-D-arabinoheptulosonate7-phosphate
E4P: erythrose 4-phosphate
IPTG: isopropyl-β-D-thiogalactopyranoside
ncRNAs: noncoding RNAs
PCA: phenazine-1-carboxylic acid
SA: shikimic acid
TCST: two-component signal transduction system
PEP: phosphoenolpyruvate
