# Supplementary figures and images for: Genetic engineering of Pseudomonas chlororaphis GP72 for the enhanced production of 2-Hydroxyphenazine
Source: Microb Cell Fact. 2016 Jul 28;15:131. doi: 10.1186/s12934-016-0529-0 (PMC4965901; doi:10.1186/s12934-016-0529-0)

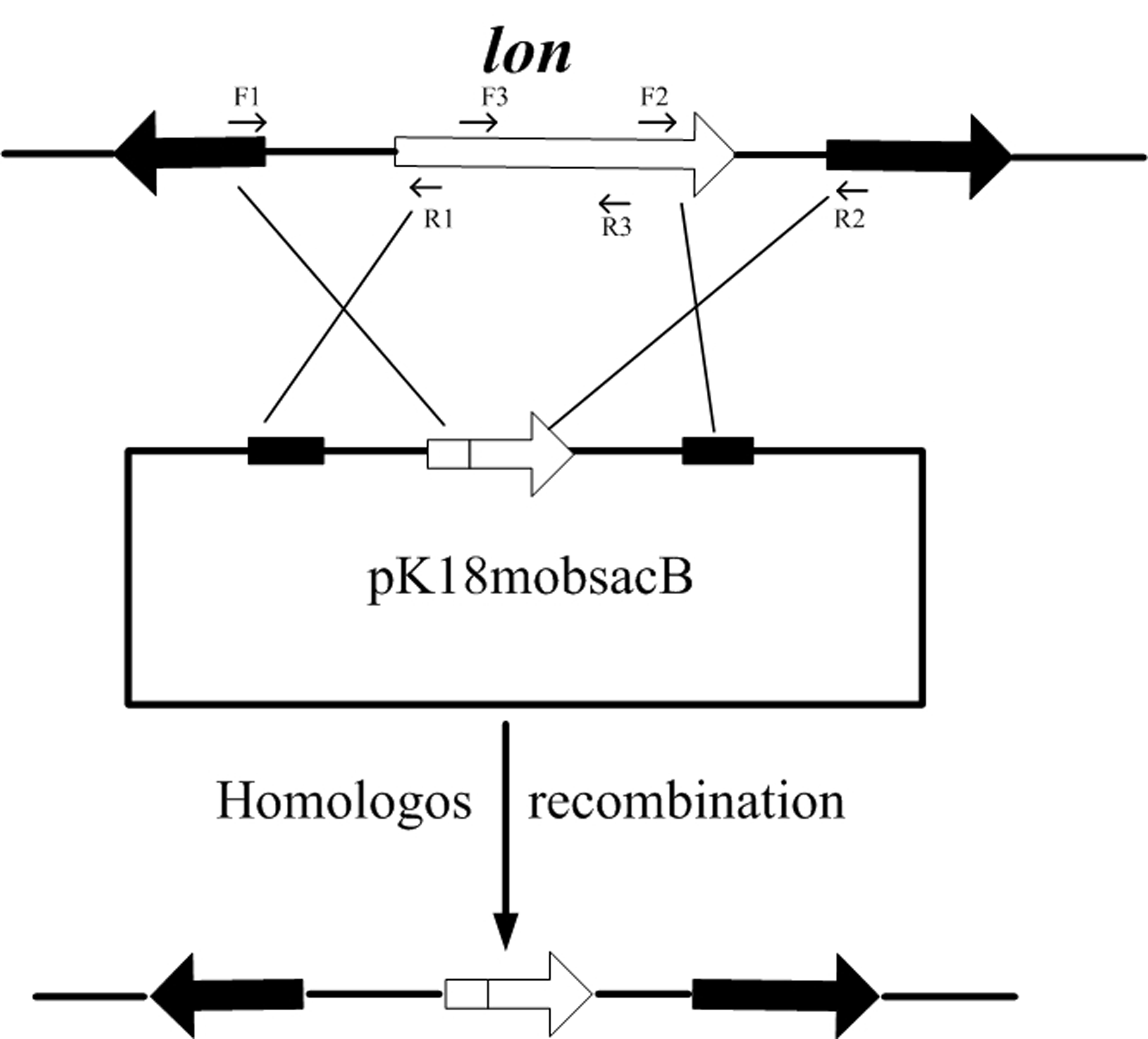

Supplement: Supplementary file 1 — 10.1186/s12934-016-0529-0 Strategy used for non-scar gene deletion. [file 12934_2016_529_MOESM1_ESM.tif]

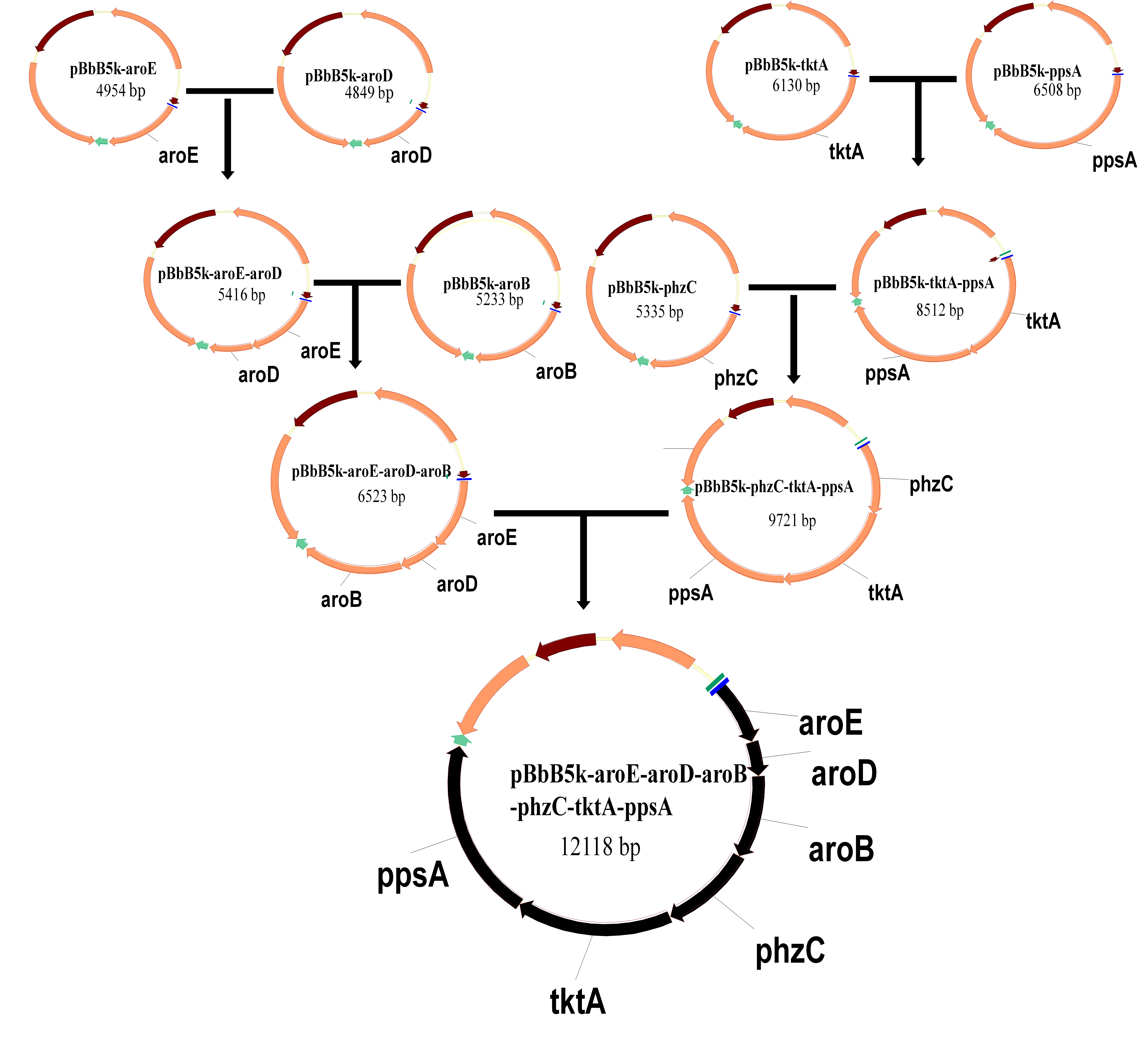

Supplement: Supplementary file 2 — 10.1186/s12934-016-0529-0 Simplified scheme of the steps required in the construction of the BglBric plasmid pBbB5K-aroE-aroD-aroB-phzC-tktA-ppsA. [file 12934_2016_529_MOESM2_ESM.tif]
