# Supplementary material for: Genetic engineering of Pseudomonas chlororaphis GP72 for the enhanced production of 2-Hydroxyphenazine
Source: Microb Cell Fact. 2016 Jul 28;15:131. doi: 10.1186/s12934-016-0529-0 (PMC4965901; doi:10.1186/s12934-016-0529-0)
Supplement: Supplementary file 3 — 10.1186/s12934-016-0529-0 Primers used in this study. [file 12934_2016_529_MOESM3_ESM.docx]

**Table S1 Primers used in this study**

| Primers | Sequence 5’ **→** 3’ | Application |
| --- | --- | --- |
| pyk-F1 | CACGAATTCCGCCGGTCCGTGAACATGTCAT | *pyk* gene deletion |
| pyk-R1 | GCAAAGACTCCTGAGTTCAAGCGCAACGAGAGG |  |
| pyk-F2 | TCAGGAGTCTTTGCGTCCCCCTGATGCAAC |  |
| pyk-R2 | AAGTCTAGACGAAGGACTGCGACAAGCCGACG |  |
| rsmE-F1 | CAACTCTAGATGCGCGACGCGGTGCTCGAT | *rsmE* gene deletion |
| rsmE -R1 | GATCTTCTCCTTGATTGCTTTGTAGGGCACCTG |  |
| rsmE -F2 | AATCAAGGAGAAGATCGCGTCATGAGCGGCCA |  |
| rsmE -R2 | GGGGAAGCTTGCTCGAAATATTGCCGATGGTG |  |
| lon-F1 | GCGTCTAGAATCCACCACCAGCCAGTCCA | *lon* gene deletion |
| lon-R1 | CTCATGGGGCACCTGCGCAATGGG |  |
| lon-F2 | AGGTGCCCCATGAGACGCAGACCTGTAG |  |
| lon-R2 | GCTAAGCTTCGCCGAGCAGGGAGAACAACA |  |
| tktA-F | AAAATGCCAAGCCGTCGTGAGCGTG | *tktA* gene cloning |
| tktA-R | TTAGTCTTCCAGCAGCTCTTCAGCCTGA |  |
| phzC-F | GGGGATGGAAGACTTACTGAAACGGG | *phzC* gene cloning |
| phzC-R | TCAAAAGGAGGCAAGGGTTGAGGTG |  |
| aroB-F | ATGCAGACACTTAAGGTCGATCTAGGCGAG | *aroB* gene cloning |
| aroB-R | TTAACCTTTAAGCTGAGCCAGGGCGC |  |
| aroD-F | ATGCGCCCTATCGTTCTGGTGCTC | *aroD* gene cloning |
| aroD-R | TCATGCCTGGGCTCCTTGAAAACG |  |
| aroE-F | ATGGATCAGTACGTCGTTTTTGGTAACCCG | *aroE* gene cloning |
| aroE-R | ATATATCAGAGCCCCAGCTGGCGCC |  |
| ppsA-F | CCCCTTGGTAGAGTACGTAGTTTCCCTC | *ppsA* gene cloning |
| ppsA-R | TTAGACCGCACCCTGCCCCTC |  |
| phzC-F | GGGGATGGAAGACTTACTGAAACGGG | phzC gene cloning |
| phzC-R | TCAAAAGGAGGCAAGGGTTGAGGTG |  |
| tktA-M1F | CGCCTACTCCGCCGA**G**TTCCCCGAGCT | First Site-directed mutagenesis for *tktA* |
| tktA-M1R | **C**TCGGCGGAGTAGGCGGAGAAGCGCTG |  |
| tktA-M2F | CCCGCTGCTGCCGGA**G**TTCCTCGGC | Second Site-directed mutagenesis for *tktA* |
| tktA-M2R | **C**TCCGGCAGCAGCGGGCCGAACGC |  |
| aroD-MF | GTGGCCGTTGCGCCGG**A**ATCCTGATCAACCCGG | Site-directed mutagenesis for *aroD* |
| aroD-MR | **T**CCGGCGCAACGGCCACGGGCGCC |  |
| ppsA-M1F | GATCATGGAAGCCGA**G**TTCCCCGAGCAACTGAA | First Site-directed mutagenesis for *ppsA* |
| ppsA-M1R | **C**TCGGCTTCCATGATCCATTGACGGATCTGGGCG |  |
| ppsA-M2F | TGACTGCTTCGAACT**T**GAGTGCCGCGCCCTC | Second Site-directed mutagenesis for *ppsA* |
| ppsA-M2R | CTCG**A**AGTTCGAAGCAGTCACGGAACGATTCGCTGATG |  |
| ppsA-M3F | CCTGGCCGAAGAGTTCCT**T**GAGTTCTTCGACGG | Third Site-directed mutagenesis for *ppsA* |
| ppsA-M3R | **A**AGGAACTCTTCGGCCAGGATCGCGTTGGAAGG |  |
| tktA--RB-F | AAGAATTCAAAAGATCTAAAAGGAGGCCATCCATGCCAAGCCGTCGT | *tktA* gene overexpression |
| tktA-RB-R | TACTCGAGTTTGGATCCTTAGTCTTCCAGCAGCTCTTCAG |  |
| phzC-RB-F | AAGAATTCAAAAGATCTAAAAGGAGGCCATCCATGGAAGACTTACTGAAACG | *phzC* gene overexpression |
| phzC-RB-R | AACTCGAGTTTGGATCCTCAAAAGGAGGCAAGGGTT |  |
| aroB-RB-F | AAGAATTCAAAAGATCTAAAAGGAGGCCATCCATGCAGACACTTAAG | *aroB* gene overexpression |
| aroB-RB-R | AACTCGAGTTTGGATCCTTAACCTTTAAGCTGAGCCAGG |  |
| aroD-RB-F | AAGAATTCAAAAGATCTAAAAGGAGGCCATCCATGCGCCCTATCGTT | *aroD* gene overexpression |
| aroD-RB-R | AACTCGAGTTTGGATCCTCATGCCTGGGCTCCTT |  |
| ppsA-RB-F | AAGAATTCAAAAGATCTAAAAGGAGGCCATCCTTGGTAGAGTACGTA | *ppsA* gene overexpression |
| ppsA-RB-R | AACTCGAGTTTGGATCCTTAGACCGCACCCTG |  |
| aroE-RB-F | AAGAATTCAAAAGATCTAAAAGGAGGCCATCCATGGATCAGTACGTCGT | *aroE* gene overexpression |
| aroE-RB-R | AACTCGAGTTTGGATCCTCAGAGCCCCAGCTGGC |  |
