# Supplementary material for: Genetic engineering of Pseudomonas chlororaphis GP72 for the enhanced production of 2-Hydroxyphenazine
Source: Microb Cell Fact. 2016 Jul 28;15:131. doi: 10.1186/s12934-016-0529-0 (PMC4965901; doi:10.1186/s12934-016-0529-0)
Supplement: Supplementary file 4 — 10.1186/s12934-016-0529-0 The accession number of genes used in this work were deposited in GenBank of NCBI. [file 12934_2016_529_MOESM4_ESM.docx]

**Table S2. The accession number of genes used in this work was deposited in GenBank of NCBI.**

| **Gene** | **Locus Tag** | **Reference Sequence Accession** |
| --- | --- | --- |
| *pykF* | MOK_RS0114440 | NZ_AHAY01000164.1 |
| *rsmE* | MOK_RS0127240 | NZ_AHAY01000292.1 |
| *rpeA* | MOK_RS0121330 | NZ_AHAY01000222.1 |
| *lon* | MOK_RS0115440 | NZ_AHAY01000170.1 |
| *phzO* | MOK_RS0116670 | NZ_AHAY01000173.1 |
| *tktA* | MOK_RS0121970 | NZ_AHAY01000233.1 |
| *ppsA* | MOK_RS0128120 | NZ_AHAY01000300.1 |
| *phzC* | MOK_RS0116645 | NZ_AHAY01000173.1 |
| *aroB* | MOK_RS0123840 | NZ_AHAY01000249.1 |
| *aroD* | MOK_RS0117670 | NZ_AHAY01000180.1 |
| *aroE* | MOK_RS0119530 | NZ_AHAY01000201.1 |
